# Metacognitive Beliefs Predict Greater Mental Contamination Severity After an Evoking Source

**DOI:** 10.3389/fpsyg.2018.01784

**Published:** 2018-10-23

**Authors:** Thomas A. Fergus, Kelsi A. Clayson, Sara L. Dolan

**Affiliations:** Department of Psychology and Neuroscience, Baylor University, Waco, TX, United States

**Keywords:** mental contamination, metacognitive beliefs, posttraumatic stress, self-regulatory executive function (S-REF) model, sexual trauma

## Abstract

Mental contamination occurs when individuals experience feelings of internal dirtiness and distress in the absence of physical contact with a contaminant. Women who experience sexual trauma frequently report mental contamination. The self-regulatory executive function (S-REF) model proposes that metacognitive beliefs contribute to the appraisal and regulation of thinking, leading to expectations that metacognitive beliefs would predict greater mental contamination severity following an evoking source. Women who reported directly experiencing sexual trauma (*N* = 102) completed self-report measures of metacognitive beliefs and covariates during an online study session, and subsequently completed a task that evoked mental contamination during a follow-up in-person study session. Metacognitive beliefs surrounding the uncontrollability and danger of thoughts, cognitive confidence, and the need to control thoughts positively correlated with mental contamination severity following the evoking source. Metacognitive beliefs surrounding the uncontrollability and danger of thoughts predicted greater mental contamination severity following the evoking source in multivariate analyses that statistically controlled for baseline mental contamination severity, trait anxiety, and overlap among the metacognitive beliefs. The present results provide preliminary support for the S-REF model as a potential framework for conceptualizing mental contamination.

## Introduction

Contamination is a near universal unpleasant feeling that can be separated into two distinct, albeit related, domains (Rachman, 2004; Coughtrey et al., 2012; Rachman et al., 2015). Contact contamination occurs when there are concerns of dirtiness, endangerment, infection, or pollution following physical contact with a source. Mental contamination—the focus of the present research—typically arises in the absence of direct physical contact with a source (Rachman, 2004; Rachman et al., 2015). Images, memories, and thoughts are common sources of mental contamination (e.g., Fairbrother et al., 2005; Herba and Rachman, 2007; Elliott and Radomsky, 2009, 2012; Rachman et al., 2012). Mental contamination ranges along a continuum of severity and, thus, typically is best conceptualized dimensionally (Radomsky et al., 2018), with prior investigations using a full range of severity scores (e.g., Elliott and Radomsky, 2009, 2013; Radomsky and Elliott, 2009; Rachman et al., 2012; Brake et al., 2018; Jacoby et al., 2018; Ojserkis et al., 2018).

Much of the extant research has focused on mental contamination in the context of obsessive-compulsive symptoms, in which individuals report experiencing internal dirtiness following ego-dystonic images or thoughts (e.g., Rachman, 2004; Elliott and Radomsky, 2009; Rachman et al., 2015). Cleansing behavior often is reported in such situations; yet, cleansing behavior ultimately contributes to the persistence of perceptions of dirtiness (Rachman et al., 2015). Despite associations with obsessive-compulsive symptoms, mental contamination likely spans across multiple forms of psychopathology (Blakey and Jacoby, 2018). The relevance of mental contamination to posttraumatic stress following sexual trauma has garnered attention, with existing study findings indicating the relatively common experience of mental contamination among women who survive sexual trauma (e.g., Fairbrother and Rachman, 2004; Fairbrother et al., 2005; Herba and Rachman, 2007; Olatunji et al., 2008; Badour et al., 2013a,b). Mental contamination subsequent to sexual trauma relates to greater posttraumatic stress symptoms (Fairbrother and Rachman, 2004; Olatunji et al., 2008; Badour et al., 2013a,b) and may be particularly relevant to understanding intrusion-related distress associated with traumatic events. For example, feelings of dirtiness may contribute to avoidant coping that maintains mental contamination and distress surrounding images, memories, and thoughts (Coughtrey et al., 2014). Indeed, following sexual trauma, women report mental contamination and avoidant coping (e.g., cleansing behavior) after trauma recall (Fairbrother and Rachman, 2004; Badour et al., 2013a). Identifying factors contributing to mental contamination holds promise for improving our understanding of posttraumatic stress following sexual trauma.

Conceptual models of mental contamination have yet to be fully developed and the purpose of the present research is to provide a preliminary examination as to whether the self-regulatory executive function (S-REF) model (Wells and Matthews, 1994) could serve as a framework for conceptualizing mental contamination. The S-REF model proposes that self-knowledge about coping guides self-regulatory efforts that ultimately maintain and worsen emotional distress (Wells and Matthews, 1994). Metacognitive beliefs (i.e., beliefs about thinking) underlie a particularly deleterious form of self-regulation known as the cognitive attentional syndrome (CAS) within the S-REF model. Threat monitoring and negatively valenced, self-referential thinking (e.g., worry) are hallmark features of the CAS (Wells and Matthews, 1994). The S-REF model has been applied to specific symptomatology, including posttraumatic stress (Wells and Sembi, 2004). The S-REF model proposes that posttraumatic stress symptoms are a normative part of an adaptation process in the acute aftermath of trauma exposure. For example, women commonly experience posttraumatic stress symptoms in the acute aftermath of sexual trauma (Shevlin et al., 2014). Mental contamination commonly is experienced by women following sexual trauma (e.g., Fairbrother and Rachman, 2004) and, thus, could be a relatively normative part of an adaptation process in the acute aftermath of sexual trauma as well.

An important consideration pertains to processes that help maintain mental contamination following sexual trauma, with the S-REF model leading to expectations that metacognitive beliefs contribute to greater mental contamination severity. For example, metacognitive beliefs activate the CAS and, thus, are responsible for “trauma-lock” (Wells and Sembi, 2004; Wells, 2009). Trauma-lock is a byproduct of the CAS that involves trauma perseveration. Supporting the potential relevance of this process to mental contamination are results that trauma reminders evoke mental contamination (Fairbrother and Rachman, 2004; Badour et al., 2013a) and re-evoking mental contamination contributes to its persistence (Coughtrey et al., 2014). Greater mental contamination severity would thus be expected to occur following trauma perseveration, which, according to the S-REF model, occurs because of metacognitive beliefs.

The S-REF model further holds that trauma-lock contributes to negative interpretations of symptoms, which often take the form of metacognitive beliefs (Wells and Sembi, 2004; Wells, 2009). For example, individuals may endorse beliefs such as “It’s not normal to keep thinking about the trauma” or “I could lose my mind if I continue to think this way” (Wells, 2009). Such beliefs commonly are termed negative metacognitive beliefs because the beliefs relate to the uncontrollability or danger of thinking (Wells, 2000). Extant research supports negative metacognitive beliefs as being particularly relevant to posttraumatic stress (Bennett and Wells, 2010; Fergus and Bardeen, 2017a), thus highlighting the possible relevance of those specific metacognitive beliefs to mental contamination following trauma exposure. Other researchers have similarly raised the possibility that negative metacognitive beliefs underlie mental contamination (e.g., “If I cannot control my repugnant, repulsive thoughts I will go crazy,” Radomsky et al., 2018). The S-REF model posits that negative metacognitive beliefs contribute to negative appraisals of symptomatology, thereby contributing to responses (e.g., worry and other CAS-relevant avoidant coping) that block emotional processing and result in greater threat perception. Underlying metacognitive beliefs are strengthened (e.g., about threat detection, danger of thinking) and trauma-lock is, thus, maintained (Wells and Sembi, 2004; Wells, 2009). The individual consequently experiences heightened emotional distress, possibly inclusive of mental contamination, because underlying metacognitive beliefs continue to fuel the process (e.g., trauma perseveration, negative interpretation of symptoms).

As noted, conceptual models of mental contamination have yet to be fully developed. Other researchers offered a preliminary cognitive conceptualization of mental contamination, which chiefly focuses on content-based self-appraisals related to responsibility and violation (Rachman et al., 2015; Radomsky et al., 2018). That conceptualization diverges from the S-REF model, as the S-REF model proposes that the impact of such appraisals on emotional distress is the result of metacognitive beliefs and the CAS (Wells, 2000). The present study sought to provide preliminary support for the S-REF model as a framework for conceptualizing mental contamination by examining metacognitive beliefs as a predictor of mental contamination severity following an evoking source among women experiencing sexual trauma. This particular sample composition was chosen because of the reviewed literature indicating that mental contamination is particularly salient for women experiencing sexual trauma.

It was expected that metacognitive beliefs would positively relate to mental contamination severity following the evoking source. In addition to examining bivariate relations, multivariate analyses examined the robustness of those relations by statistically controlling for the effects of theoretically relevant covariates. Examined covariates included trait anxiety, disgust proneness, and posttraumatic stress symptom severity, each of which has shown relevance to mental contamination in prior research (e.g., Badour et al., 2014; Ojserkis et al., 2018). Including covariates in multivariate analyses allowed for an examination as to the incremental explanatory power of metacognitive beliefs in accounting for mental contamination severity. Multivariate analyses also statistically controlled for mental contamination severity before the evoking source to ensure observed effects captured something beyond baseline severity. Following from extant data that beliefs surrounding the danger and uncontrollability of thinking are metacognitive beliefs particularly relevant to posttraumatic stress (Bennett and Wells, 2010; Fergus and Bardeen, 2017a), as well as other indices of emotional distress (e.g., Spada et al., 2008), those metacognitive beliefs were expected to emerge as particularly relevant to mental contamination severity within multivariate analyses.

## Materials and Methods

### Participants

A total of 713 undergraduate women at a private Southern United States university were screened for potential participation. Eligibility criteria were women who reported personally experiencing sexual trauma on the Life Events Checklist for *DSM-5* (LEC-5; Weathers et al., 2013a). More precisely, eligibility involved women who endorsed directly experiencing sexual assault or another unwanted or uncomfortable sexual experience on the LEC-5. A broad definition of sexual trauma was used following findings that women may resist endorsing experiencing sexual trauma when questions contain stigmatized terminology, such as “rape” (e.g., Resnick et al., 1993). A total of 206 women were eligible for participation (28.9% of the total screened sample), a percentage consistent with the lifetime prevalence of sexual trauma found in undergraduate samples of women (Frazier et al., 2009).

Of those 206 eligible participants, 102 participated in the lab-based session (49.5% of eligible participants). The average age of those 102 women was 19.4 years (*SD* = 3.1, range 18–38), with 56.9% self-identifying as White, 16.7% as Latina, 9.8% as multi-racial, 8.8% as Black, 5.9% as Asian, and 1.9% as “other” ethnicity or race. There were no significant age (*t*_(204)_ = 0.76, *p* = 0.451) or ethnoracial ($\chi_{\left(5\right)}^{2}$ = 1.99, *p* = 0.851) differences between women who were eligible and did versus did not participate. There also were no significant differences on any of the study variable scores reported below between women who were eligible and did versus did not participate (magnitude of *t*_(204)_ ranged from 0.66 to 1.31, *p*s > 0.193).

### Measures

#### Metacognitions Questionnaire-30 (MCQ-30; Wells and Cartwright-Hatton, 2004)

The MCQ-30 is a 30-item short form of the 65-item MCQ (Cartwright-Hatton and Wells, 1997). Both MCQ versions assess the same five metacognitive beliefs: (a) positive beliefs about worry; (b) negative beliefs about the uncontrollability and danger of thoughts; (c) cognitive confidence; (d) need for control; and (e) cognitive self-consciousness. The distinctiveness of the five metacognitive beliefs of the MCQ-30 has since been replicated (Spada et al., 2008; Fergus and Bardeen, 2017b). MCQ-30 items are rated using a 4-point scale (ranging from 1 to 4). The MCQ-30 scales show approximately 5-week test-retest correlation coefficients ranging from 0.59 to 0.79 (Wells and Cartwright-Hatton, 2004). The MCQ-30 scales showed adequate to good internal consistency in the present study (Cronbach’s αs ranging from 0.76 to 0.91).

#### State Trait Inventory for Cognitive and Somatic Anxiety (STICSA; Ree et al., 2008)

The STICSA is a 21-item self-report measure of anxiety using separate state and trait versions. In regards to trait anxiety, participants rate the degree to which each item indicates how they “generally feel.” STICSA items are rated using a 4-point scale (ranging from 1 to 4). The STICSA assesses cognitive and somatic anxiety, with a total score derived by summing the 21 item scores. Higher scores reflect greater trait anxiety. The STICSA shows approximately 7-week test-retest correlation coefficients of 0.60 and 0.66 (Ree et al., 2008). The STICSA showed good internal consistency in the present study (α = 0.88).

#### Disgust Propensity and Sensitivity Scale-Revised (DPSS-R; van Overveld et al., 2006)

The DPSS-R is a 16-item self-report measure of disgust proneness, conceptualized as the propensity to experience disgust and negative appraisals of disgust. DPSS-R items are rated using a 5-point scale (ranging from 1 to 5). A 12-item version that improves upon the factorial validity of the measure was used (Fergus and Valentiner, 2009). Higher scores reflect greater disgust proneness. The DPSS-R shows approximately 8-week test-retest correlation coefficients of 0.69 and 0.67 (van Overveld et al., 2006). The DPSS-R showed good internal consistency in the present study (α = 0.85).

#### PTSD Checklist for *DSM-5* (PCL-5; Weathers et al., 2013b)

The PCL-5 is a 20-item self-report measure that assesses posttraumatic stress symptoms following PTSD criteria in the *DSM-5* (American Psychiatric Association, 2013). PCL-5 items are endorsed using a 5-point scale (ranging from 0 to 4). A total score is derived by summing intrusion, hyperarousal, avoidance, and negative alterations in cognition and mood symptoms over the past month. Higher scores reflect greater symptom severity. The PCL-5 shows an approximately 1-week test-retest correlation coefficient of 0.82 (Blevins et al., 2015). The PCL-5 showed good internal consistency in the present study (α = 0.93). Participants completed the PCL-5 in relation to the LEC-5 event that currently bothered them the most^1^.

#### State Mental Contamination Scale (SMCS; Lorona et al., 2018)

The SMCS is a 15-item self-report measure that assesses state mental contamination and was developed to parallel the items of the trait measure of mental contamination known as the Vancouver Obsessive-Compulsive Inventory-Mental Contamination Scale (VOCI-MC; Radomsky et al., 2014). Lorona et al. (2018) reworded 15, of the 20, items from the VOCI-MC so that the timeframe of the SMCS items reflected the present moment. The remaining five VOCI-MC items were not conducive to rewording to the present moment and were dropped from the item pool. SMCS items are rated using a 5-point scale (ranging from 1 to 5). A total score is derived by summing the 15 item scores. Higher scores indicate greater state mental contamination and the SMCS showed good internal consistency in the present study (α = 0.94).

### Procedure

The local institutional review board approved the study protocol. Separate informed consent processes were completed before the online and lab-based session. The LEC-5 was completed online to determine study eligibility. Participants also completed the MCQ-30, STICSA, DPSS-R, and PCL-5 during the online session. The self-report measures were completed during a separate session to ensure the study activities in the lab-based session did not inadvertently influence responses to the self-report measures. Moreover, completing the self-report measures during the separate online session helped reduce the likelihood that responses to those activities influenced responses in the lab-based session. There was an average of 22 days (*SD* = 16) between the online and the individual lab-based session^2^. Each eligible participant was invited to participate in the lab-based session through an e-mail, of which, as noted, only a subset of eligible participants signed-up for the later study session. Eligible participants who attended the lab-based session initially completed an item asking about current feelings of dirtiness rated using a 0 to 100 scale, with 100 representing the greatest severity, to assess baseline mental contamination severity. Participants completed that same item again following the evoking task for purposes of a manipulation check (e.g., Elliott and Radomsky, 2009).

For the evoking task, participants completed the “dirty-kiss” task (Elliott et al., 2008) in which they listened to an audio recording through headphones that instructed them to imagine attending a party with a friend. At the party, participants imagined receiving a non-consensual kiss from a male described as possessing disgusting qualities. The recording ends with the friend asking, “How did you end up kissing that guy?” and participants then take off the headphones. This task has been used in prior research to evoke mental contamination (e.g., Elliott and Radomsky, 2012). Immediately following task completion, participants completed the SMCS. Participants then completed items related to the ease of imagining, vividness, and realism of the scenario using a 0 to 100 scale, with higher scores indicating greater ease imagining, vividness, and realism. Ratings indicated a high degree of ease imagining (*M* = 84.25, *SD* = 22.09), vividness (*M* = 82.41, *SD* = 19.22), and realism (*M* = 75.74, *SD* = 25.41) in the present study. Participants were then debriefed. Participants received partial course credit for their participation in both the online and lab-based session.

## Results

### Preliminary Analyses

A paired-samples *t*-test was used to examine the effectiveness of the dirty-kiss task by comparing feelings of dirtiness from before, *M* = 18.32, *SD* = 22.14, and after, *M* = 65.95, *SD* = 28.82, the task. That analysis indicated a significant increase in dirtiness ratings, *t*_(101)_ = 15.03, *p* < 0.001, and the effect was large in magnitude, Cohen’s *d* = 1.86. The task had its intended effect.

Descriptive statistics and zero-order correlations among the study variables are presented in Table 1. The maximum magnitude values for skewness (baseline feelings of dirtiness: 1.09) and kurtosis (PCL-5: 0.94) of the study variables were below levels typically considered elevated (i.e., | 2| ; Bandalos, 2018). As such, the distributions of scores did not appear to substantively deviate from normality. The metacognitive beliefs generally significantly intercorrelated with the covariates, save for baseline feelings of dirtiness only correlating with cognitive confidence. As predicted, metacognitive beliefs generally positively correlated with mental contamination severity following the evoking source (i.e., SMCS scores). Those correlations were small-to-moderate in magnitude. Associations with mental contamination severity following the evoking source were found in relation to negative metacognitive beliefs, cognitive confidence, and the need for control. Because positive metacognitive beliefs and cognitive self-consciousness did not correlate with mental contamination severity following the evoking source, the positive metacognitive beliefs and cognitive self-consciousness scales of the MCQ-30 were dropped from multivariate analyses (e.g., Thielsch et al., 2015). In addition, among the covariates, only trait anxiety and baseline feelings of dirtiness correlated with mental contamination severity following the evoking source. As such, disgust proneness and posttraumatic stress symptoms were dropped as covariates from multivariate analyses.

**Table 1:** Descriptive statistics and zero-order correlations.

Variable	Mean	*SD*	1	2	3	4	5	6	7	8	9
(1) MCQ-30-P	11.96	4.81	–								
(2) MCQ-30-N	13.34	4.49	0.37^∗∗^	–							
(3) MCQ-30-CC	11.24	4.55	0.13	0.37^∗∗^	–						
(4) MCQ-30-NC	12.38	3.96	0.43^∗∗^	0.55^∗∗^	0.22^∗^	–					
(5) MCQ-30-CSC	15.20	4.51	0.20^∗^	0.39^∗∗^	0.10	0.54^∗∗^	–				
(6) STICSA-Trait	40.74	9.70	0.29^∗∗^	0.61^∗∗^	0.49^∗∗^	0.32^∗∗^	0.27^∗∗^	–			
(7) DPSS-R	28.52	7.60	0.31^∗∗^	0.36^∗∗^	0.20^∗^	0.26^∗∗^	0.05	0.44^∗∗^	–		
(8) PCL-5	21.11	15.45	0.28^∗∗^	0.39^∗∗^	0.24^∗^	0.27^∗∗^	0.32^∗∗^	0.38^∗∗^	0.15	–	
(9) Baseline dirtiness	18.32	22.14	0.03	−0.05	0.26^∗∗^	0.18	0.13	0.19	0.05	0.07	–
(10) SMCS	21.38	13.65	0.13	0.32^∗∗^	0.23^∗^	0.25^∗^	0.05	0.24^∗^	0.08	0.19	0.32^∗∗^

*N = 102. ^∗∗^p < 0.01, ^∗^p < 0.05 (two-tailed). MCQ, Metacognitions Questionnaire (P, Positive; N, Negative; CC, Cognitive Confidence; NC, Need for Control; CSC, Cognitive Self-Consciousness); STICSA, State-Trait Inventory of Cognitive and Somatic Anxiety; DPSS-R, Disgust Propensity and Sensitivity Scale-Revised; PCL-5, PTSD Checklist for DSM-5; SMCS, State Mental Contamination Scale.*

### Regression Analyses

A hierarchical multiple linear regression was used to examine the unique variance accounted for by metacognitive beliefs in mental contamination severity following the evoking source (i.e., SMCS scores). The retained covariates (trait STICSA, baseline feelings of dirtiness) were entered into Block 1 of the model. The retained metacognitive variables were entered into subsequent blocks in descending order based upon the magnitude of zero-order correlations with mental contamination severity following the evoking source. As such, negative metacognitive beliefs from the MCQ-30 were entered into Block 2, need for control from the MCQ-30 was entered into Block 3, and cognitive confidence from the MCQ-30 was entered into Block 4. The maximum variance inflation factor (VIF) among the predictors in the regression analysis was 2.31, well below conventional guidelines for indicating problems with multicollinearity (>10; Cohen et al., 2003). The maximum Cook’s *D* value was 0.08, well below conventional guidelines for indicating the presence of an overly influential case on the regression model (>1.0; Cohen et al., 2003). The maximum Mahalanobis distance value was 14.95 and, thus, there were no values at or above the respective critical value for indicating multivariate outliers (χ^2^_(5)_ = 20.52, *p* < 0.001; Mertler and Vannatta, 2005).

The variance accounted for in mental contamination severity following the evoking source and standardized beta weights from the regression analysis are presented in Table 2. As shown, the covariates collectively accounted for 14% of variance in mental contamination severity in Block 1. Adding negative metacognitive beliefs to the model in Block 2 accounted for an additional 8% of variance in mental contamination severity. Adding metacognitive beliefs related to need for control and cognitive confidence in Block 3 and Block 4, respectively, did not explain additional variance in mental contamination severity. In Block 4, baseline feelings of dirtiness and negative metacognitive beliefs were the only significant statistical predictors.

**Table 2:** Hierarchical regression results examining predictors of mental contamination severity following evoking source.

	State mental contamination scale											
	Block 1 Results			Block 2 Results			Block 3 Results			Final Block Results		
	Δ*R*^2^	β	*t*	Δ*R*^2^	β	*t*	Δ*R*^2^	β	*t*	Δ*R*^2^	β	*t*
Block 1	0.14^∗∗^											
STICSA-Trait		0.19	1.96		−0.05	0.40		−0.05	0.50		−0.06	0.50
Baseline Dirtiness		0.29^∗∗^	3.02		0.35^∗∗^	3.75		0.35^∗∗^	3.40		0.34^∗∗^	3.40
Block 2				0.08^∗∗^								
MCQ-30-N					0.36^∗∗^	3.15		0.36^∗∗^	2.68		0.36^∗∗^	2.59
Block 3							<0.01					
MCQ-30-NC								0.01	1.26		0.01	0.02
Block 4										<0.01		
MCQ-30-CC											0.04	0.41

*N = 102. ^∗∗^p < 0.01, ^∗^p < 0.05 (two-tailed). STICSA, State-Trait Inventory of Cognitive and Somatic Anxiety; MCQ, Metacognitions Questionnaire (N, Negative; NC, Need for Control; CC, Cognitive Confidence).*

## Discussion

The present study sought to provide a preliminary examination of the S-REF model (Wells and Matthews, 1994) as a framework for conceptualizing mental contamination by investigating metacognitive beliefs as predictors of mental contamination severity. An S-REF model applied to posttraumatic stress (Wells and Sembi, 2004) was the selected framework for the present study given the frequent occurrence of mental contamination following sexual trauma. Women who experienced sexual trauma completed a self-report measure of metacognitive beliefs and later completed a task that evoked mental contamination. Consistent with study predictions, metacognitive beliefs generally positively correlated with mental contamination severity following the evoking task. In bivariate analysis, metacognitive beliefs related to the uncontrollability and danger of thoughts, cognitive confidence, and need for control shared small-to-moderate correlations with mental contamination severity. However, only negative metacognitive beliefs (i.e., uncontrollability and danger of thoughts) related to mental contamination severity in multivariate analyses, suggesting, as predicted, that those metacognitive beliefs are particularly relevant to mental contamination.

The association between negative metacognitive beliefs and mental contamination severity is notable because it was found even while statistically controlling for baseline mental contamination severity, trait anxiety, and interrelations among other metacognitive beliefs. Disgust proneness and posttraumatic stress symptom severity were not included as covariates in multivariate analyses, as those two variables unexpectedly did not correlate with mental contamination severity following the evoking source in the present study. That pattern of findings stands in contrast to prior findings that disgust and posttraumatic stress symptoms are associated with changes in feelings of dirtiness from before to after an evoking source among women experiencing sexual trauma (Badour et al., 2014). Sample composition could be one reason for the discrepant findings, as Badour et al. (2014) used a narrower group of respondents experiencing sexual trauma (i.e., women reporting sexual trauma exposure and denied history of physical assault) than the present study. Assessment method could be another reason for discrepant findings, as Badour et al. assessed mental contamination via feelings of dirtiness alone. Although that assessment method is common (e.g., Elliott and Radomsky, 2009), and was included as a manipulation check in the present study, Radomsky et al. (2014) contend that mental contamination is more fully represented through aspects other than feelings of dirtiness. The state measure of mental contamination used in the present study follows Radomsky et al.’s contention via conceptualizing mental contamination as broader than feelings of dirtiness alone (Lorona et al., 2018). Although tenable possibilities, future research examining the contribution of disgust proneness and posttraumatic stress symptoms to *in-vivo* experiences of mental contamination appears warranted before firmer conclusions about those interrelations are drawn.

Negative metacognitive beliefs were expected to be the metacognitive beliefs particularly relevant to mental contamination following from extant findings linking those metacognitive beliefs to posttraumatic stress (Bennett and Wells, 2010; Fergus and Bardeen, 2017a). In the context of posttraumatic stress, beliefs about the uncontrollability and danger of thoughts putatively lead to threatening interpretations of symptoms that contribute to emotional distress (Wells and Sembi, 2004; Wells, 2009). Images, memories, and thoughts are common sources of mental contamination (e.g., Fairbrother et al., 2005; Herba and Rachman, 2007; Elliott and Radomsky, 2009, 2013; Rachman et al., 2012). Following from the S-REF model, negative interpretations of symptoms would be expected to increase the likelihood of unwanted images, memories, and thoughts occurring (Wells and Sembi, 2004; Wells, 2009) and, thereby, re-evoke mental contamination. Prior research indicates that re-evoking mental contamination contributes to its persistence (Coughtrey et al., 2014). Additionally, negative metacognitive beliefs could contribute to the engagement in avoidant behavior in an attempt to regulate thoughts, with that behavior ultimately blocking emotional processing and maintaining distress (Wells, 2000). Cleansing behavior is a common type of avoidant behavior reported in the context of mental contamination (Rachman et al., 2015) and future research should seek to examine whether negative metacognitive beliefs are associated with cleansing behavior following a mental contamination provocation.

Conceptual models of mental contamination have yet to be fully developed, with existing cognitive conceptualizations emphasizing the role of negative appraisals in relation to mental contamination (Rachman et al., 2015; Radomsky et al., 2018). Whereas such conceptualizations do not preclude the consideration of metacognitive beliefs, existing examinations of the relevance of cognitive variables to mental contamination have tended to focus on content-based self-appraisals related to responsibility and violation (e.g., Radomsky and Elliott, 2009; Elliott and Radomsky, 2013). The S-REF model would lead to predictions that mental contamination is not the result of such content-based self-appraisals (e.g., “I am pathetic, weak, hopeless,” Radomsky et al., 2018), but is the result of metacognitive beliefs and the CAS (Wells, 2000). Content-based self-appraisals, unfortunately, were unexamined. Future research that concurrently examines content-based self-appraisals and metacognitive beliefs will aid in elucidating the degree to which those variables incrementally contribute to our understanding of mental contamination.

Additional support for the S-REF model as a tenable framework for conceptualizing mental contamination could come from future research findings that content-based self-appraisals do not account for unique variance in mental contamination severity once statistically controlling for metacognitive beliefs, such as the uncontrollability and danger of thoughts, or the CAS. Such patterns of findings have emerged in prior studies examining obsessive-compulsive symptoms (e.g., Myers et al., 2009; Solem et al., 2010). It is important to note that content-based self-appraisals can initiate self-regulatory efforts in the form of the CAS (Wells, 2000). It is thus possible that the relationship between content-based self-appraisals and mental contamination depends upon metacognitive beliefs or the CAS serving as moderators. Indeed, extant research supports that possibility when considering the relation between content-based self-appraisals and the frequency of ego-dystonic intrusive thoughts (Fergus and Wu, 2010). Another possibility is that the impact of the types of beliefs on mental contamination differs across time, which could be examined in future longitudinal research.

Future research supporting the relevance of metacognitive beliefs to mental contamination would point to potential treatment strategies when seeking to reduce mental contamination. For example, such patterns of findings may point to a focus on the metacognitive mode in which intervention strategies chiefly target how one relates to cognitive events (Wells, 2000). A greater focus on the metacognitive mode, rather than the object mode, in which the content of appraisals are evaluated for their accuracy, could be preferred when seeking to reduce mental contamination. The present results indicate the relevance of negative metacognitive beliefs to mental contamination. Intervention strategies relevant to mitigating negative metacognitive beliefs about the uncontrollability and danger of thoughts include verbal reattribution, behavioral experiments, and detached mindfulness (Wells, 2009). Detached mindfulness seeks to promote the metacognitive mode by having individuals consider themselves as an observer separate from their thoughts to facilitate suspension of conceptual processing and the alteration of metacognitive beliefs (Wells, 2009). Current treatment efforts for mental contamination are in their relative infancy (Coughtrey et al., 2013) and future research may seek to examine the usefulness of metacognitive intervention strategies in the reduction of mental contamination severity.

Study limitations must be considered. As previously reviewed, the present study focused on women experiencing sexual trauma given that mental contamination is particularly salient for these individuals. Indeed, the evoking task produced a large increase in mental contamination severity among study participants. However, the inclusion criteria was broad in that other types of trauma exposure were not restricted. In addition, there was lack of available information on the nature of the sexual trauma and the frequency of sexual trauma was not assessed. The generality of the present findings to women experiencing sexual trauma would thus be strengthened through determining study eligibility following a more in-depth assessment of sexual trauma exposure. A large number of eligible participants did not participate in the lab-based session. Although eligible participants who did versus did not participate in the lab-based session did not differ on demographic information or study variable scores, it is possible that the subset of participants who attended the lab-based session differed in some unknown ways from participants who did not attend that study session. Trauma exposure is common among college students (Frazier et al., 2009) and, yet, the generality of the findings would be further strengthened by examining the relation between metacognitive beliefs and mental contamination among community respondents. The present study was adequately powered (1 – β = 0.80) to detect small-sized effects in the examined regression model (Cohen’s *f*^2^ ≈0.08; Aiken and West, 1991), as determined using a *post hoc* power analysis (Faul et al., 2009). Future research replicating and extending the findings with larger samples nonetheless appears warranted (e.g., Schönbrodt and Perugini, 2013).

Studies commonly examine mental contamination among women (e.g., Elliott and Radomsky, 2009, 2013; Radomsky and Elliott, 2009; Badour et al., 2013a,b, 2014). Nevertheless, men experience mental contamination as well (e.g., Coughtrey et al., 2012). A limitation of the dirty-kiss task is that it is most appropriate for women (Elliott and Radomsky, 2009). Future research should thus seek to use alternative methods for evoking mental contamination (De Putter et al., 2017) in order to replicate the present findings among samples consisting of both sexes. By using alternative evoking sources and other samples, future research can help address whether metacognitive beliefs generally account for mental contamination severity or whether the impact of those beliefs seems most relevant in the context of posttraumatic stress. The study methods precluded the consideration of causal relations between metacognitive beliefs and mental contamination. Future longitudinal and experimental research is needed to address if negative metacognitive beliefs causally influence mental contamination. The self-report measures of the statistical predictors were completed, on average, 22 days before the completion of the lab-based session, which was done to ensure the study activities in the lab-based session did not inadvertently influence responses to the self-report measures or vice versa. As discussed, scores on the self-report measures of the statistical predictors have evidenced stability estimates considered moderate to high for trait variables (e.g., Roberts et al., 2008) in prior research. Nonetheless, interrelations between mental contamination and the other study variables may have been impacted by the gap between study sessions.

The examined variables, collectively, accounted for about 22% of the variance in mental contamination severity, with negative metacognitive beliefs accounting for about 8% of unique variance. Additional variables to consider in future research include content-based self-appraisals (e.g., Radomsky and Elliott, 2009) and markers of the CAS (e.g., rumination, worry; Wells, 2000). It is possible that variables from other metacognitive models could be useful in accounting for additional variance in mental contamination severity. Links between mental contamination and obsessive-compulsive symptoms (e.g., Rachman, 2004; Rachman et al., 2015) highlight the possibility that variables from the metacognitive model of obsessive-compulsive symptoms (Wells, 2000) warrant consideration. Potentially relevant variables from that model include thought-fusion beliefs, beliefs about rituals, and stop signals.

Limitations notwithstanding, the present results provide support for the relevance of metacognitive beliefs to mental contamination. Negative metacognitive beliefs surrounding the uncontrollability and danger of thoughts accounted for unique variance in mental contamination severity following an evoking source. Continued support for a link between metacognitive beliefs and mental contamination could further support the S-REF model as a potential framework for conceptualizing mental contamination, and may ultimately lead to the use of intervention strategies that target those beliefs when seeking to reduce mental contamination.

## Ethics Statement

This study was carried out in accordance with the recommendations of the Committee for Protection of Human Subjects at Baylor University with written informed consent from all subjects. All subjects gave written informed consent in accordance with the Declaration of Helsinki. The protocol was approved by the Committee for Protection of Human Subjects at Baylor University.

## Author Contributions

TF, KC, and SD conceptualized the study. KC oversaw the data collection. TF completed the study analyses and wrote the first draft of the manuscript. KC and SD provided the feedback on that draft. TF incorporated that feedback into the submitted version, with KC and SD agreeing on the submitted version.

## Conflict of Interest Statement

The authors declare that the research was conducted in the absence of any commercial or financial relationships that could be construed as a potential conflict of interest.

## Footnotes

1 Approximately half of the sample (i.e., 48%) indicated that sexual trauma was the most bothersome LEC-5 event and no other LEC-5 event was endorsed as the most bothersome by more than 10% of the sample. SMCS scores following the evoking task did not significantly differ based upon whether sexual trauma was the most distressing LEC-5 event (*t*_(100)_ = 1.32, *p* = 0.192). Negative metacognitive beliefs continued to share an association with SMCS scores when including the most distressing LEC-5 event (sexual trauma versus non-sexual trauma) as a covariate (β = 0.34, *p* = 0.015, in final block of regression analysis). The most distressing LEC-5 event (sexual trauma versus non-sexual trauma) did not moderate the association between negative metacognitive beliefs and SMCS scores (β = −0.18, *p* = 0.590, for the interaction term).

2 Days between study sessions did not correlate with any of the study variables (magnitude of *r*s ranging from 0.01 to 0.12, *p*s > 0.252).
